# The effects of smoking on genotoxic and histopathologic damage in exfoliated oral epithelial cells and the periodontium: A cross-sectional study

**DOI:** 10.1097/MD.0000000000033140

**Published:** 2023-02-22

**Authors:** Begum Alkan, Pinar Koroglu-Aydin

**Affiliations:** a Private Practice of Periodontology, Istanbul, Turkey (formerly Department of Periodontology, Faculty of Dentistry, Istanbul Medipol University, Istanbul, Turkey); b Department of Histology and Embryology, Faculty of Medicine, Halic University, Istanbul, Turkey.

**Keywords:** epithelial cells, histopathology, micronuclei, periodontitis, smoking

## Abstract

Smoking negatively affects the prognosis of periodontal disease by impairing tissue healing. While micronucleus is the most popular parameter for demonstrating DNA damage, inflammatory cell and vascular densities are the most evaluated parameters for determining histopathologic changes in the periodontium. This study aimed to study the effects of periodontitis and cigarette smoking on genotoxic changes in exfoliated oral epithelial cells and histopathologic changes in periodontal tissue. A cross-sectional study was conducted between November 2018 and July 2019 at a dental university hospital in Turkey, and registered as NCT05484765. Eighty systemically healthy subjects were divided into four groups according to periodontal status and smoking habits: 20 smokers with generalized periodontitis (SGP), 20 nonsmokers with generalized periodontitis (NGP), 20 smokers with healthy periodontium (SHP), and 20 nonsmokers with healthy periodontium (NHP). For each study participant, full-mouth clinical periodontal parameters (CPPs) were measured, smear samples were taken from buccal and gingival mucosa, and periodontal tissue was biopsied from the maxillary molars. Cytogenetic and histopathologic assays (primary and secondary outcomes) were conducted using Feulgen reaction and hematoxylin-eosin staining, respectively. The mean CPPs of healthy periodontium groups were lower than generalized periodontitis groups. No significant differences were found between other groups regarding CPPs. Buccal micronuclei counts in groups decreased with the highest to lowest counts occurring in the order SGP > SHP > NGP > NHP. Gingival micronuclei counts in groups decreased from SGP > SHP > NGP = NHP. The most intense inflammatory cell and vascular densities occurred in SGP and NGP groups, respectively; and the mildest values were in healthy periodontium groups. Histopathological damage score decreased significantly by group in order SGP > NGP > SHP > NHP. The synergy arising from the combination of smoking and periodontitis exposures exacerbates genotoxic and histopathologic damage in oral cells and the periodontium.

## 1. Introduction

Periodontitis, which causes tooth loss via the deformation of the tissues supporting the teeth, is a public health problem characterized by low-grade chronic inflammation.^[1]^ Although the primary etiological factor for the condition is microbial dental plaque, various risk factors such as an unhealthy lifestyle or systemic disease can reduce resistance to disease and increase the severity of tissue destruction.^[2]^ Among the lifestyle-related factors that can contribute to periodontitis, smoking is the most important preventable risk factor^[3]^ after microbial dental plaque. Clinical studies have found that smokers are more prone to periodontitis than nonsmokers, and smoking increases the severity and rate of periodontal diseases.^[2,3]^ Moreover, smoking adversely affects the outcome of both non-surgical and surgical periodontal treatments; therefore, adjunctive therapeutic approaches have been proposed to treat periodontitis in smokers.^[3]^ However, smoking does not always result in periodontitis and the mechanisms underlying the effect of smoking on the severity and progression of periodontitis have not been fully elucidated.

During mitosis, loss of chromatin from chromosomal DNA is manifested by extra-small, abnormal nuclear structures called micronuclei.^[4–7]^ Of the available cytogenetic analyses, the micronucleus assay is one of the most popular methods for detecting structural and numerical nuclear abnormalities due to its reliability and simplicity.^[6–9]^ This genotoxic biomonitoring assay makes it possible to provide evidence for the biological assessment of disease susceptibility, diagnosis, and staging in subjects exposed to environmental risk factors.^[4,6]^ The oral mucosa provides a barrier against potential carcinogens, and a cytogenetic analysis of exfoliated oral epithelial cells is the preferred method for detecting genotoxic effects caused by inhaled agents such as cigarettes.^[6,8,10]^ As inflammatory conditions such as periodontitis may induce DNA damage,^[5,11]^ a careful evaluation of the micronuclei counts in subjects with generalized periodontitis is particularly relevant to standardizing the buccal and gingival micronuclei counts in assays and increasing their diagnostic use.

Clinical signs and symptoms of gingival inflammation tend to decrease in smokers,^[3,12,13]^ and this finding can be confused with gingival health.^[14–16]^ Moreover, a histopathologic approach is not used in routine periodontal disease assessment in clinical practice as the tissue biopsy must be examined in a histology laboratory.^[17]^ Therefore, a routine diagnosis of periodontal diseases is typically evaluated using full-mouth clinical periodontal parameters (CPPs) such as probing depth (PD) measurements and bleeding on probing (BOP) detection.^[18]^ However, during clinical examination, nonstandard probing forces can cause significant calculation errors in the evaluation of PD and BOP.^[19]^ Conversely, histopathologic evaluations can help to identify the destructive stages of risk factors such as smoking at the tissue and cellular levels^[17]^ and can be used to assess two critical parameters involved in the evolution of periodontal inflammation – inflammatory cell and vascular densities. In addition, various damage scoring methods used in the histopathological analysis of tissue can also play a valuable role in reaching an accurate outcome. This cross-sectional study aimed to evaluate the effects of heavy cigarette smoking and generalized periodontitis on local genotoxic damage to exfoliated oral epithelial cells as well as histopathologic damage to the periodontium. We hypothesized that the genotoxic and histopathologic damage would be increased in smokers with generalized periodontitis.

## 2. Materials and Methods

### 2.1. Study design and setting

This was a single-center, investigator-blinded, cross-sectional study. Work was conducted at the Department of Periodontology in Istanbul, Turkey, between November 2018 and July 2019. The study procedure was conducted according to the guidelines of the Declaration of Helsinki and approved by the Ethics Committee of the university (Protocol code: 10840098 – 604.01.01 – E.47596 and 10840098 – 604.01.01 – E.4759; date of approval: October 30, 2018). Data from six nonsmoking participants with healthy periodontium from our previous studies were used in this study as well.^[20]^ After the study was verbally explained to eligible subjects, those willing to participate signed a written, informed consent form. Personal, identifiable information about the subjects was kept confidential. The study was registered at clinicaltrials.gov (NCT05484765) and was reviewed following the Strengthening the Reporting of Observational Studies in Epidemiology guidelines.^[21]^

### 2.2. Participants

Eighty systemically healthy subjects, both male and female, were enrolled in the study. Participants were recruited from patients who came to our department for periodontal examinations between November 2018 and July 2019. Diagnoses of clinically healthy periodontium and generalized periodontitis stages III-IV/grades B-C were performed according to the 2017 World Workshop on the Classification of Periodontal and Peri-implant Diseases and Conditions.^[18]^

Subjects were separated into four groups: smokers with generalized periodontitis (SGP; n = 20), nonsmokers with generalized periodontitis (NGP; n = 20), smokers with clinically healthy periodontium (SHP; n = 20), and nonsmokers with clinically healthy periodontium (NHP; n = 20). The following criteria needed to be met to be considered a periodontitis subject: the presence of generalized periodontitis,^[18]^ the presence of at least 20 teeth, the presence of a molar with an indication of gingivectomy, crown lengthening or extraction with a PD ≥ 5 mm, clinical attachment loss ≥ 5 mm, and the presence of at least ten teeth with a PD ≥ 5 mm. The following criteria indicated a clinically healthy periodontium: a healthy and intact periodontium,^[18]^ no PD and BOP.

The inclusion criteria for the study were: self-reporting as systemically healthy, aged between 18 and 65 years, and the presence of at least five teeth in each quadrant. In addition, participants needed to undergo either gingivectomy operations, crown lengthening procedures, or tooth extractions as part of their treatment so periodontal tissue samples could be obtained from all subjects. All smokers had used cigarettes for at least 5 years with a daily consumption of ≥ 20. A willingness to participate in the study and give written informed consent was also necessary. Exclusion criteria were as follows: the presence of a known medical condition that could possibly bias the results (e.g., pregnancy, lactation, cardiovascular diseases, diabetes mellitus, chronic inflammatory diseases, rheumatoid arthritis, hepatitis, tuberculosis, cancers, previous radiotherapy or chemotherapy); having a medical condition requiring antibiotic prophylaxis for dental treatment; the presence of systemic medication taken within the previous 6 months (e.g., anticonvulsants, antibiotics, or corticosteroids); excessive alcohol consumption; currently undergoing nicotine replacement therapy; consuming tobacco products other than cigarettes; those with fixed orthodontic appliances; and history of periodontal therapy.

### 2.3. Clinical procedure

At the first appointment, all subjects filled out a questionnaire assessing age, gender, and educational level. The education program proposed by the target participant’s age was adjusted to consider three levels: primary education (1–8 years of schooling), secondary education (9–11 years of schooling), and tertiary education level (12 years or more of schooling). A clinical evaluation of periodontal disease was conducted according to international standards.^[22]^ The CPPs, including plaque index,^[23]^ PD (measured after resistance was felt in the apical region of the sulcus), BOP (detected after 30 seconds of probe insertion into the sulcus), and clinical attachment loss (calculated the sum of PD and gingival recession measurement), were recorded from six sites per tooth from each subject using a William’s periodontal probe (Hu-Friedy, Chicago, IL) by the same experienced periodontist researcher (B.A.). The sample collection and histopathologic evaluation procedures were performed as in our previous report.^[20]^ Briefly, smear samples were collected from the attached gingival mucosa of the upper premolar-molar area and the buccal mucosa of the matching cheek using two separate sterile cytobrushes. Samples were fixed on glass slides. Biopsy samples were taken from the vestibular area of the maxillary molars during tooth extraction or crown lengthening using a 15C surgical blade and fixed in 10% formalin until analysis. During microscopic inspection, each subject rinsed their mouth before samples were obtained to reduce the possibility of artifacts through chromatin remainders.^[24]^

### 2.4. Laboratory procedure

To evaluate the exfoliated oral epithelial cells containing micronuclei counts (the primary outcome), the Feulgen reaction (Bio-Optica, Milan, Italy) was performed on all smear samples as described by Thomas et al^[10]^ Cells were placed in a fixative for nuclear staining and analysis using Schiff’s reagent (Bio-Optica). Micronuclei in the buccal and gingival smear samples were analyzed by observing them in oil immersion at 1000× magnification. One thousand cells were checked, and the count of micronuclei present was assessed.

Histopathologic damage (the secondary outcome) was evaluated by focusing on inflammatory cell and vascular densities. In addition to these parameters, the histopathological damage score, which is the mean of cell infiltration, cellular components, vascularization, hyperemia, necrosis, and pycnotic nuclei in the tissue, was evaluated using a modified semi-quantitative scale ranging from 0 to 3 (0: none, 1: mild, 2: moderate, and 3: intense). Tissue samples were fixed in paraffin wax, serially sectioned at a 4 µm thickness, stained with hematoxylin-eosin (Biognost, Zagreb, Croatia), and examined at 400× magnification. All smear and tissue samples were assayed concurrently and in the same laboratory by the same blinded investigator (P.K.).

### 2.5. Bias

The evaluation of each participant’s systemic health status, dental history, and smoking habits was based on self-reported information regardless of official medical records. One researcher (B.A.) collected the data and samples from the participants while another researcher (P.K.) undertook the laboratory investigations. To avoid bias, five sample areas were randomly selected during the histologic examinations.

### 2.6. Study size

The sample size was decided based on similar studies in this field and determined using G*Power (Franz Faul Universidad, Kiel, Germany) version 3.1.9.4, assuming an alpha significance level of 0.05, a beta value of 0.1, and 90% power. As there were four study groups and a need for 20 participants per group, eighty subjects were included in the study.

### 2.7. Statistical methods

The Number Cruncher Statistical System 2007 (NCSS, Kaysville, UT) software program was used for all statistical analyses. Descriptive statistics (mean, standard deviation, median) were used to evaluate the data. The one-way analysis of variance test was used to compare the means of three or more normally distributed parameters. The Bonferroni test was used to determine the significant differences in pairwise comparisons between the groups. The Pearson chi-square test was used to compare qualitative data. Significance was evaluated at the *P* < .01 and *P* < .05 levels.

## 3. Results

**Figure 1.**
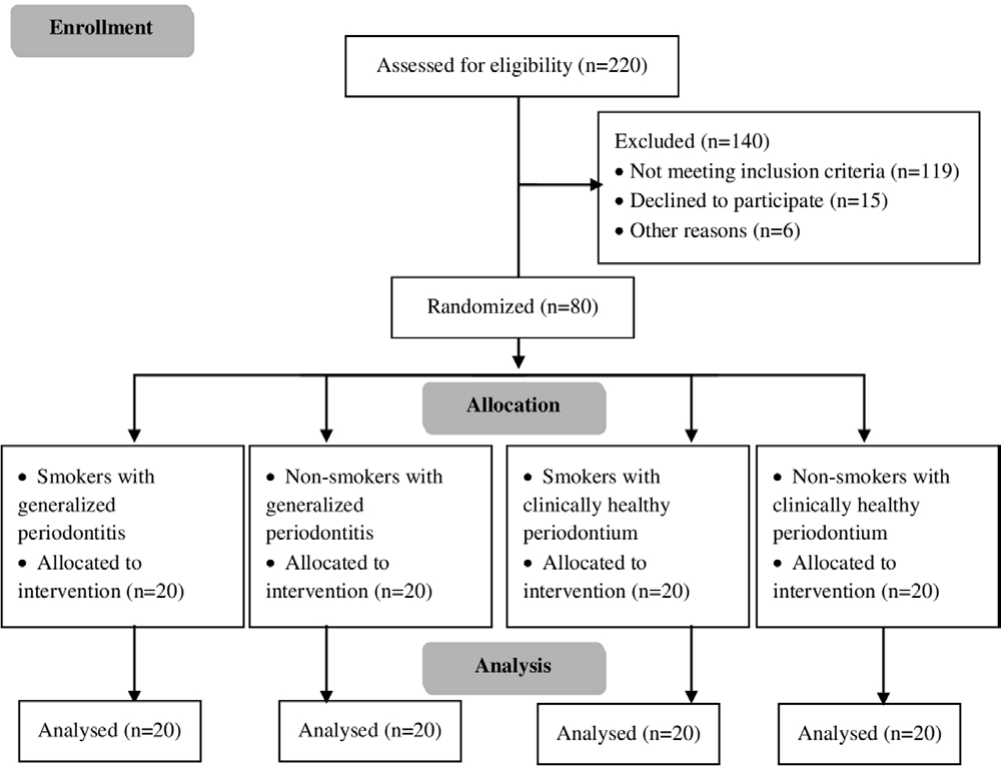
Flow diagram showing the study protocol.

A flow diagram showing the study protocol is shown in Figure 1. No subjects left the study, and all data were included in the statistical analysis. The distributions of age, gender, and educational level were significantly different between groups and are listed in Table 1. The average age was lower in the NHP group compared to the other three groups, and the average age was lower in the SHP group in comparison to the generalized periodontitis groups (*P* < .01). The percentage of females was highest in the NHP group, while the SGP group had the highest percentage of males.

**Table 1:** Age, gender, and education by study group.

	SGP	NGP	SHP	NHP	*P*
	n = 20	n = 20	n = 20	n = 20	
Age (yr)†					
Mean ± standard deviation (median)	45.4 ± 8.85 (43.5)	50.1 ± 11.3 (52.5)	37.05 ± 10.47 (38)	28.6 ± 8.02 (25)	.001*
Gender, n (%)‡					
Male	17 (85)	9 (45)	11 (55)	4 (20)	.001*
Female	3 (15)	11 (55)	9 (45)	16 (80)	
Education, n (%)‡					
1–8 yr	14 (70)	19 (95)	13 (65)	3 (15)	.001*
9–11 yr	5 (25)	1 (5)	2 (10)	6 (30)	
12 yr or more	1 (5)	0 (0)	5 (25)	11 (55)	

NGP = nonsmokers with generalized periodontitis, NHP = nonsmokers with healthy periodontium, SGP = smokers with generalized periodontitis, SHP = smokers with healthy periodontium.

* *P* < .01.

† One way analysis of variance test.

‡ Pearson chi-square test.

The mean, standard deviation, and median values of the CPPs are given in Table 2. The CPPs averages showed significant differences according to group. Both the NHP and SHP groups exhibited significantly lower values in all CPPs than the NGP and SGP groups (*P* < .01). In both the generalized periodontitis and healthy periodontium groups, the CPPs results were similar between nonsmokers and smokers (*P* > .05).

**Table 2:** Mean ± standard deviation and (median) of clinical periodontal parameters by study group.

	SGP	NGP	SHP	NHP	*P*
	n = 20	n = 20	n = 20	n = 20	
Plaque index†	1.15 ± 0.32 (1.08)	1.01 ± 0.26 (1.02)	0.43 ± 0.36 (0.25)	0.51 ± 0.39 (0.5)	.001**
Probing depth, mm†	4.91 ± 0.6 (4.92)	4.64 ± 0.78 (4.62)	2.04 ± 0.34 (1.88)	2.29 ± 0.32 (2.2)	.001**
Bleeding on probing, %†	74.9 ± 11.9 (77.5)	87.85 ± 6.6 (85.1)	1.72 ± 1.88 (1)	2.32 ± 2.03 (2)	.001**
Clinical attachment loss, mm†	5.37 ± 0.8 (5.47)	4.92 ± 0.97 (4.85)	2.15 ± 0.35 (2)	2.38 ± 0.32 (2.5)	.001**

NGP = nonsmokers with generalized periodontitis, NHP = nonsmokers with healthy periodontium, SGP = smokers with generalized periodontitis, SHP = smokers with healthy periodontium.

** *P* < .01.

† One way analysis of variance test.

**Figure 2.**
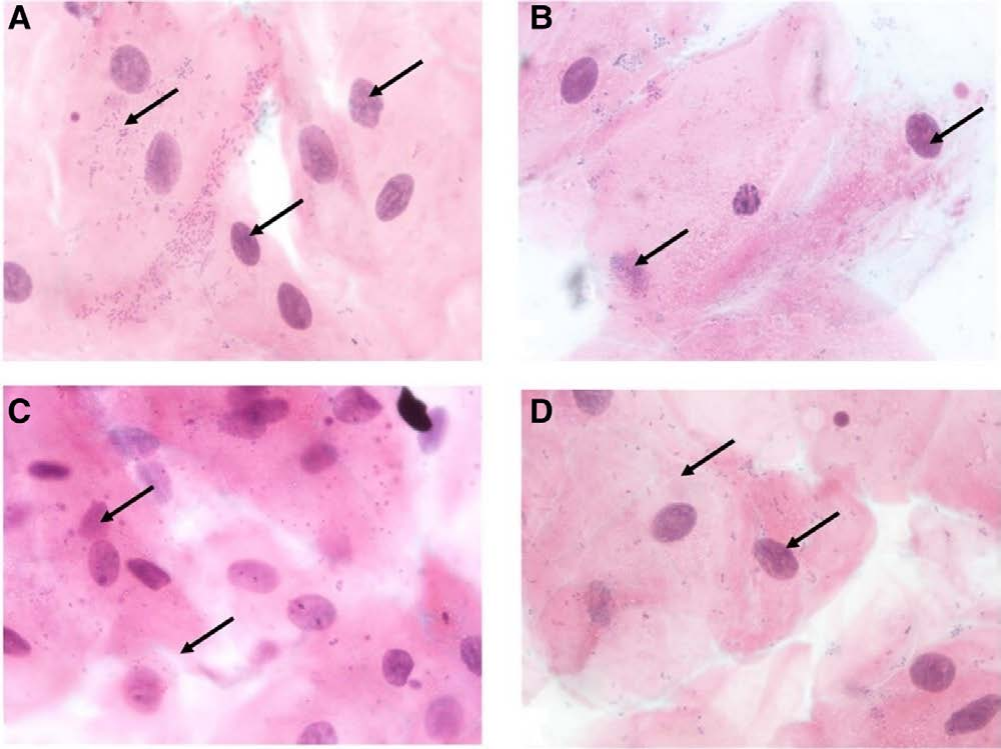
Buccal mucosa smear samples from all groups: micronucleus (→). Photomicrographs of exfoliated epithelial cells belong to (A) smokers with generalized periodontitis, (B) nonsmokers with generalized periodontitis, (C) smokers with clinically healthy periodontium, and (D) nonsmokers with clinically healthy periodontium (Feulgen staining, 1000×).

**Figure 3.**
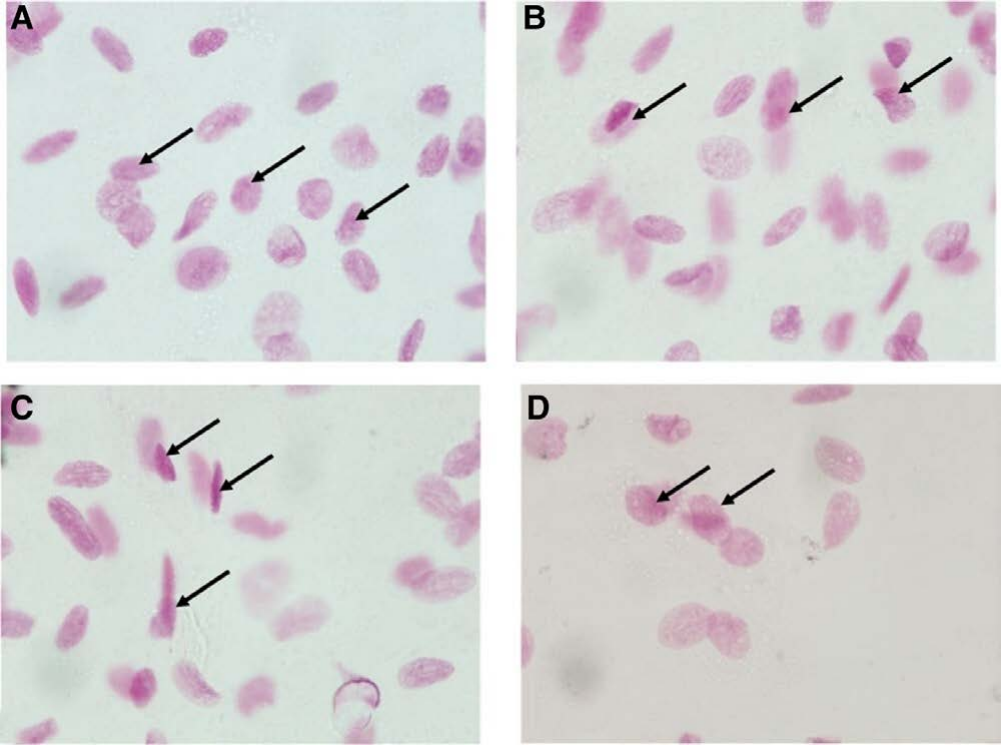
Gingival mucosa smear samples from all groups: micronucleus (→). Photomicrographs of exfoliated epithelial cells belong to (A) smokers with generalized periodontitis, (B) nonsmokers with generalized periodontitis, (C) smokers with clinically healthy periodontium, and (D) nonsmokers with clinically healthy periodontium (Feulgen staining, 1000×).

**Figure 4.**
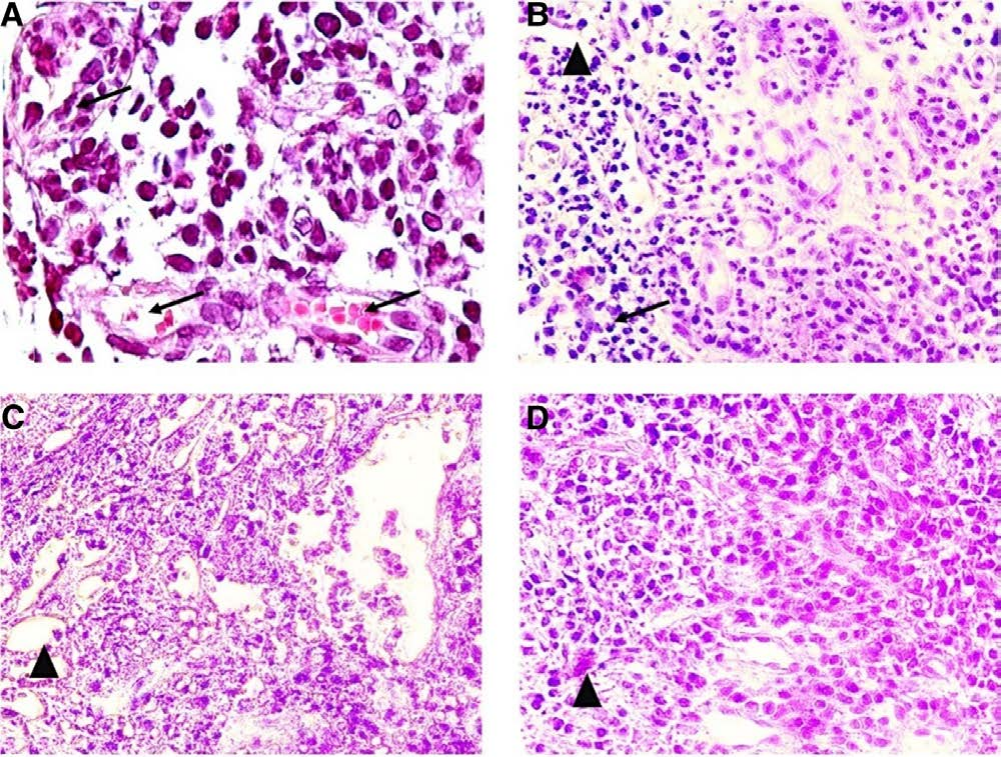
Periodontal tissue samples from all groups: inflammatory cell density (→), and vascular density (▲). Photomicrographs of periodontal tissues belong to (A) smokers with generalized periodontitis, (B) nonsmokers with generalized periodontitis, (C) smokers with clinically healthy periodontium, and (D) nonsmokers with clinically healthy periodontium (Hematoxylin and eosin staining, 40×).

The distribution of laboratory results between the study groups is outlined in Table 3. Buccal micronuclei counts decreased significantly by group in the order: SGP > SHP > NGP > NHP (Fig. 2). Gingival micronuclei counts similarly decreased with SGP > SHP > NGP = NHP (Fig. 3). The histological view of periodontal tissue samples is shown in Figure 4. The highest inflammatory cell density was found in the SGP group, while the lowest counts were found in both healthy periodontium groups. The most intense vascular density was found in the NGP group, and the mildest was in the NHP and SHP groups. Histopathological damage score decreased significantly by group in order: SGP > NGP > SHP > NHP.

**Table 3:** Distribution of laboratory findings among study groups.

	SGP	NGP	SHP	NHP	*P*
	n = 20	n = 20	n = 20	n = 20	
Buccal micronuclei counts†					
Mean ± standard deviation (median)	7 ± 1.89 (7.5)	2.05 ± 1.5 (2)	5.3 ± 1.22 (5)	0.15 ± 0.37 (0)	.001**
Gingival micronuclei counts†					
Mean ± standard deviation (median)	7.3 ± 1.75 (7.5)	0.6 ± 0.6 (0.5)	4.8 ± 1.32 (5)	0.2 ± 0.52 (0)	.001**
Inflammatory cell density, n (%)‡					
None	–	–	10 (50)	20	.001**
Mild	5 (25)	14 (70)	10 (50)	–	
Moderate	12 (60)	6 (30)	–	0	
Intense	3 (15)	–	–	–	
Vascular density, n (%)‡					
None	3 (15)	–	15 (75)	19 (95)	.001**
Mild	17 (85)	4 (20)	5 (25)	1 (5)	
Moderate	–	16 (80)	–	–	
Intense	–	–	–	–	
Histopathologic damage score†					
Mean ± standard deviation (median)	1.46 ± 0.23 (1.5)	0.72 ± 0.16 (0.75)	0.45 ± 0.24 (0.42)	0.01 ± 0.04 (0)	.001**

NGP = nonsmokers with generalized periodontitis, NHP = nonsmokers with healthy periodontium, SGP = smokers with generalized periodontitis, SHP = smokers with healthy periodontium.

** *P* < .01.

† One way analysis of variance test.

‡ Pearson chi-square test.

## 4. Discussion and conclusion

As far as we know, the present study is the first study to evaluate DNA damage in the oral epithelium by computing micronuclei counts in exfoliated buccal and gingival mucosal epithelial cells and assessing histopathologic status in the periodontium of smokers and nonsmokers with periodontitis and those of subjects who were clinically periodontally healthy and without systemic disease.

Our results found that all CPPs values for both healthy periodontium groups were lower than those for the generalized periodontitis groups, irrespective of smoking status. Studies have reported that smokers with periodontitis have lower gingival bleeding indices than nonsmokers with periodontitis owing to nicotine’s vasoconstrictive property.^[12,25]^ We believe that the BOP score did not differ significantly between periodontitis groups as a result of the pressure exerted by the periodontal probe while placed it in the constricted periodontal pocket in the smokers group, and the excessive PD caused by the severe periodontal inflammation.

Tissue damage in inflammatory periodontal pathologies may occur through the induction of proinflammatory cytokines and DNA.^[26]^ Inflammatory processes are associated with cell division and may cause chromosomal damage that stimulates apoptosis during mitosis.^[27]^ Increased frequency of apoptosis may then exert genotoxic effects related to the initiation of a malignant transformation process.^[27]^ DNA damage reflects events associated with progressive stages of carcinogenicity. In this study, we have sought to determine whether periodontitis exerts genotoxic effects and, if so, whether its diagnosis is suitable for use as a biomarker. The evaluation of micronuclei counts is useful for understanding the genotoxic effects of various environmental lifestyle factors, including tobacco use, on exfoliated oral epithelial cells. However, several contradictory results have been reported for regarding micronuclei counts in cells obtained from different sampling sites in patients with periodontitis and in tobacco studies.^[4,5,24,28]^ Therefore, this study investigated the effects of periodontitis and smoking on DNA damage by examining buccal and gingival smear samples.

The micronuclei counts in exfoliated buccal mucosal epithelial cells were higher in groups with periodontitis than in clinically healthy groups. These counts were also higher in smokers than in nonsmokers. These results are in line with those of several periodontitis studies^[11,24,28,29]^ and smoking studies^[9,24,28,30–32]^ that have reported higher micronuclei counts in exfoliated buccal mucosal epithelial cells of periodontal patients and smokers than in controls, suggesting that periodontitis and smoking may affect baseline micronuclei counts in exfoliated buccal mucosal epithelial cells. For example, Bloching et al^[24]^ observed higher micronuclei counts in exfoliated buccal mucosal epithelial cells in subjects with periodontitis, particularly those with periodontitis who use tobacco. Similarly, a review of clinical trials published in 2018 observed higher micronuclei counts in the exfoliated oral epithelial cells of smokers than in those of nonsmokers, which the authors attributed to tobacco’s cytotoxic and genotoxic effects.^[32]^ Contrary to our results, however, several studies^[33,34]^ have found no association between periodontal disease status and micronuclei counts in exfoliated buccal mucosal epithelial cells.

We also found that the micronuclei counts in exfoliated gingival mucosal epithelial cells was highest in the SGP group and lowest in the nonsmoker groups. Several clinic-based case-control studies have assessed the genotoxic effect of periodontitis on gingival epithelial cells. While some studies^[4–7,27,35]^ found other types of nuclear damage such as binucleation and apoptosis in epithelial cells, to be associated with periodontal inflammation, they observed no significant relationship between micronucleus numbers and periodontal inflammation. However, an in vitro study^[36]^ reported significantly increased micronuclei counts in human gingival fibroblasts following exposure to nicotine for 24 hours. Similarly, Tadin et al^[7]^ reported that smoking was associated with high micronuclei counts in exfoliated gingival mucosal epithelial cells. One pilot study^[5]^ reported that an increase in the micronuclei counts in exfoliated epithelial cells obtained from marginal gingival smears was not associated with periodontitis alone, although an exponential increase in the micronuclei counts was observed when the subjects had a concomitant tobacco habit. Silva et al^[37]^ observed that the micronuclei counts in epithelial cells obtained from diseased and healthy gingival areas of 10 subjects were significantly higher in diseased gingival areas. The authors concluded that moderate, chronic periodontitis disrupted the genetic integrity of gingival epithelial cells.

Our results indicate that tobacco use and periodontitis each exert distinct genotoxic effects on exfoliated oral epithelial cells from different regions of the mouth and that their effects are more pronounced when both exposures exist concurrently. This suggests that exfoliated buccal mucosal epithelial cells are more susceptible to genotoxic damage than exfoliated gingival mucosal epithelial cells. This is perhaps due to the limited DNA repair capacity of buccal epithelial cells, which may make them more suitable for use as genotoxicity biomarkers.^[8]^ However, this result does not fully reflect the long-term genotoxic effects, given the higher turnover rate of non-keratinized gingival epithelial cells.^[4]^ It has also been reported that other cytogenetic damage may be masked^[27,33]^ as a result of increased cell apoptosis as periodontal infection progresses.^[37]^ Moreover, studies have shown that the sampling area,^[32]^ preferred staining techniques,^[9,30,32,34]^ different microscope types,^[9,34]^ and deficiencies in scoring standardization in laboratory assays^[33]^ may all result in differences in the assessment of micronuclei counts.

In our study, individuals who both smoked and had periodontitis were more prone to increased micronuclei counts than individuals for whom only one of these factors was present. This may be attributed to the synergistic effect of smoking and periodontitis, which exacerbates genomic damage in exfoliated buccal and gingival mucosal epithelial cells. Nuclear anomalies in exfoliated oral epithelial cells and their relationship to oral diseases should be further investigated to better assess the prognosis of periodontal diseases and to promote the importance of good oral health. Further research, including intervention studies, is thus needed to evaluate the relationship between periodontal diseases and genotoxic interactions given that observation of the evolution of genotoxic effects may be helpful in the clinical assessment of disease prognosis.

The progression of periodontal pathology involves two critical parameters: inflammatory cell and vascular densities. While our inflammatory cell density results are similar to several studies,^[38,39]^ they are inconsistent with the results of other investigations that either did not find a significant difference between study groups^[13,25,40]^ or found inflammatory cell density more intense in nonsmokers.^[12,41]^ The vascular density results in our study align with observations from the existing literature.^[25,42–44]^ However, some studies^[12,13,45,46]^ have observed no significant differences in vascular characteristics among their study groups. In one recent study^[25]^ that compared gingival biopsies taken from an area with periodontitis with those from another area that showed no clinical signs of gingival inflammation (each taken from smokers and nonsmokers), the authors reported that while small differences in inflammatory cell density and cellular components were found in the periodontitis tissues of both the nonsmokers and smokers, the nonsmoker’s vascular density was significantly higher than that of the smokers. They also reported that clinically healthy reference sites presented similar vascular densities in smokers and nonsmokers. Regarding the histopathological damage score, none of the reviewed studies expressed the relationship between smoking and periodontal tissues with this calculation. Few histopathology studies have formed groups similar to those used in our study, making it difficult to draw clear comparisons between our results and those of other studies due to the differences in the initial criteria for periodontal status and cigarette use, the different approaches used to select tissue samples from different areas of the oral cavity, and the various methods used to measure and evaluate changes in histopathologic structures.

The present study supports the findings of previous investigations and offers further evidence for the impact of heavy tobacco consumption on oral structures. This study’s main limitations include possible recall bias on the part of the participants with respect to their smoking habits and the inability to generalize the cause-effect relationship between smoking and periodontitis due to the study’s cross-sectional design. The participants’ dietary habits, the dental biomaterials used in restorative procedures, and X-ray exposures to which participants had recently been exposed were not evaluated within the scope of the exclusion criteria. Another limitation is that the participants’ age and gender demographic characteristics could not be balanced due to the limited study period. This may affect the findings and impede generalization problems. Several studies^[7,10,24]^ have reported that a higher age and being male is positively associated with a micronuclei count; however, Konopacka^[47]^ study found that neither age nor gender affected micronuclei count level.

Among the present study’s strengths is the assessment of the subjects’ periodontal status using the new classification proposed by the most recent workshop of the American Academy of Periodontology and the European Federation of Periodontology held in Chicago in 2017. Therefore, we are confident that our findings lend robust support to the relationship between generalized periodontitis and our microscopic findings.

In this study, we confirmed that generalized periodontitis combined with heavy smoking is associated with poorer prognosis than smoking or periodontitis alone due to the disruption of tissue-healing mechanisms. Clinical studies, including topics that apply microbiological and immunological parameters, are required to evaluate the mechanisms underlying the effects of smoking on oral tissue. We determined that increases in micronuclei counts in exfoliated oral epithelial cells and undesirable histopathologic features were higher in the SGP group than in the other groups. We believe that monitoring micronuclei counts levels and clinical parameters at various stages of treatment may be beneficial in evaluating disease prognosis.

Our findings reveal that heavy smoking and generalized periodontitis can independently cause genotoxic and histopathological damage, but that their harmful effects are exacerbated in combination. This result highlights the importance of regulating heavy smoking habits to increase the likelihood that periodontitis treatment will be successful. Compared to tissue biopsy, the micronucleus test is an easy, noninvasive, and inexpensive procedure for examining epithelial reactions in the oral cavity. However, both observational and long-term prognostic studies with large sample sizes are required if this test is to be considered a suitable biomarker for periodontal inflammation or successful periodontal treatment.

## Author contributions

**Conceptualization:** Begum Alkan, Pinar Koroglu-Aydin.

**Data curation:** Begum Alkan.

**Formal analysis:** Begum Alkan.

**Funding acquisition:** Begum Alkan, Pinar Koroglu-Aydin.

**Investigation:** Begum Alkan, Pinar Koroglu-Aydin.

**Methodology:** Begum Alkan, Pinar Koroglu-Aydin.

**Project administration:** Begum Alkan.

**Resources:** Begum Alkan, Pinar Koroglu-Aydin.

**Software:** Begum Alkan.

**Supervision:** Begum Alkan, Pinar Koroglu-Aydin.

**Validation:** Begum Alkan, Pinar Koroglu-Aydin.

**Visualization:** Begum Alkan, Pinar Koroglu-Aydin.

**Writing – original draft:** Begum Alkan.

**Writing – review & editing:** Begum Alkan.

## References

[1] Kassebaum NJ Bernabe E Dahiya M. Global burden of severe periodontitis in 1990-2010: a systematic review and meta-regression. J Dent Res. 2014;93:1045–53. 25261053 10.1177/0022034514552491 PMC4293771

[2] Calsina G Ramon JM Echeverria JJ. Effects of smoking on periodontal tissues. J Clin Periodontol. 2002;29:771–6. 12390575 10.1034/j.1600-051x.2002.290815.x

[3] Nociti F Casati M Duarte P. Current perspective of the impact of smoking on the progression and treatment of periodontitis. Periodontol 2000. 2015;67:187–210. 25494601 10.1111/prd.12063

[4] D’Agostini F Calcagno E Micale RT. Cytogenetic analysis of gingival epithelial cells, as related to smoking habits and occurrence of periodontal disease. Int J Hyg Environ Health. 2013;216:71–5. 22357102 10.1016/j.ijheh.2012.01.005

[5] Shee F Pralhad S Natarajan S. Cellular and biochemical changes in different categories of periodontitis: a patient-based study. J Int Soc Prev Community Dent. 2020;10:341–9. 32802782 10.4103/jispcd.JISPCD_42_20 PMC7402257

[6] Tadin A Gavic L Roguljic M. Assessment of cytogenetic damage to exfoliated gingival cells in patients with chronic generalized periodontitis. Acta Clin Croat. 2021;60:209–15. 34744270 10.20471/acc.2021.60.02.06 PMC8564832

[7] Tadin A Gavic L Roguljic M. Nuclear morphological changes in gingival epithelial cells of patients with periodontitis. Clin Oral Investig. 2019;23:3749–57. 10.1007/s00784-019-02803-5 30685794

[8] Holland N Bolognesi C Kirsch-Volders M. The micronucleus assay in human buccal cells as a tool for biomonitoring DNA damage: the HUMN project perspective on current status and knowledge gaps. Mutat Res. 2008;659:93–108. 18514568 10.1016/j.mrrev.2008.03.007

[9] Sharma S Rai S Misra A. Quantitative and qualitative analysis of micronuclei in the buccal mucosal cells of individuals associated with tobacco. MAMC J Med Sci. 2018;4:12–7.

[10] Thomas P Holland N Bolognesi C. Buccal micronucleus cytome assay. Nat Protoc. 2009;4:825–37. 19444240 10.1038/nprot.2009.53

[11] Bastos-Aires D Azevedo A de Lurdes Pereira M. Preliminary study of micronuclei levels in oral exfoliated cells from patients with periodontitis. J Dent Sci. 2013;8:200–4.

[12] Jalayer Naderi N Semyari H Elahinia Z. The impact of smoking on gingiva: a histopathological study. Iran J Pathol. 2015;10:214–20. 26351487 PMC4539769

[13] Sreedevi M Ramesh A Dwarakanath C. Periodontal status in smokers and nonsmokers: a clinical, microbiological, and histopathological study. Int J Dent. 2012;2012:1–10. 10.1155/2012/571590 PMC3296295 22505904

[14] Preber H Bergstrom J. Occurrence of gingival bleeding in smoker and non-smoker patients. Acta Odontol Scand. 1985;43:315–20. 3878653 10.3109/00016358509046515

[15] Bergstrom J Persson L Preber H. Influence of cigarette smoking on vascular reaction during experimental gingivitis. Scand J Dent Res. 1988;96:34–9. 3422504 10.1111/j.1600-0722.1988.tb01405.x

[16] Lie MA Timmerman MF van der Velden U. Evaluation of 2 methods to assess gingival bleeding in smokers and non-smokers in natural and experimental gingivitis. J Clin Periodontol. 1998;25:695–700. 9763323 10.1111/j.1600-051x.1998.tb02509.x

[17] Gillett IR Johnson NW Curtis MA. The role of histopathology in the diagnosis and prognosis of periodontal diseases. J Clin Periodontol. 1990;17:673–84. 2262579 10.1111/j.1600-051x.1990.tb01053.x

[18] Caton JG Armitage G Berglundh T. A new classification scheme for periodontal and peri-implant diseases and conditions - Introduction and key changes from the 1999 classification. J Periodontol. 2018;89(Suppl 1):S1–8. 29926946 10.1002/JPER.18-0157

[19] Greenstein G Caton J Polson AM. Histologic characteristics associated with bleeding after probing and visual signs of inflammation. J Periodontol. 1981;52:420–5. 6973623 10.1902/jop.1981.52.8.420

[20] Alkan B Koroglu P. Effects of amoxicillin on gingival biopsies and oral smears: a cross-sectional study. Niger J Clin Pract. 2021;24:233–9. 33605914 10.4103/njcp.njcp_660_19

[21] Vandenbroucke JP von Elm E Altman DG.; STROBE initiative. Strengthening the reporting of observational studies in epidemiology (STROBE): explanation and elaboration. Ann Intern Med. 2007;147:W163–94. 17938389 10.7326/0003-4819-147-8-200710160-00010-w1

[22] Papapanou PN Sanz M Buduneli N. Periodontitis: consensus report of workgroup 2 of the 2017 world workshop on the classification of periodontal and peri-implant diseases and conditions. J Periodontol. 2018;89(Suppl 1):S173–82. 29926951 10.1002/JPER.17-0721

[23] Loe H. The gingival index, the plaque index and the retention index systems. J Periodontol. 1967;38:610–6. 10.1902/jop.1967.38.6.610 5237684

[24] Bloching M Reich W Schubert J. Micronucleus rate of buccal mucosal epithelial cells in relation to oral hygiene and dental factors. Oral Oncol. 2008;44:220–6. 17434785 10.1016/j.oraloncology.2007.02.002

[25] Schmidt JC Jajjo E Berglundh T. Periodontitis lesions in smokers and non-smokers. Eur J Oral Sci. 2020;128:196–203. 32304269 10.1111/eos.12693

[26] Sezer U Cicek Y Canakci CF. Increased salivary levels of 8-hydroxydeoxyguanosine may be a marker for disease activity for periodontitis. Dis Markers. 2012;32:165–72. 22377732 10.3233/DMA-2011-0876 PMC3826812

[27] Brandão P Gomes-Filho I Cruz S. Can periodontal infection induce genotoxic effects? Acta Odontol Scand. 2015;73:219–25. 25428625 10.3109/00016357.2014.982705

[28] Reich W. The Ames and Micronucleus Test as a Means of Early Detection of Carcinogenic Exposure in the Oral Cavity and their Relation to Dental Status [Der Ames- und Mikrokerntest als Möglichkeiten zur Früherkennung kanzerogener Exposition in der Mundhöhle und ihre Beziehung zum dentalen Status] [Doctoral dissertation]. Germany: Department of Dentistry, Oral and Maxillofacial Medicine, Martin Luther University Halle-Wittenberg; 2005. Available at: https://opendata.uni-halle.de/bitstream/1981185920/9290/1/prom.pdf.

[29] Zamora-Perez AL Ortiz-Garcia YM Lazalde-Ramos BP. Increased micronuclei and nuclear abnormalities in buccal mucosa and oxidative damage in saliva from patients with chronic and aggressive periodontal diseases. J Periodontal Res. 2015;50:28–36. 24666368 10.1111/jre.12175

[30] Devadoss S Raveendranath MC Kathiresan TS. Genotoxic effect of various forms of tobacco on oral buccal mucosa and nuclear changes as a biomarker. J Pharm Bioallied Sci. 2021;13(Suppl 2):S1141–8. 35017946 10.4103/jpbs.jpbs_185_21 PMC8686962

[31] Pop AM Coros R Stoica AM. Early diagnosis of oral mucosal alterations in smokers and e-cigarette users based on micronuclei count: a cross-sectional study among dental students. Int J Environ Res Public Health. 2021;18:13246. 34948855 10.3390/ijerph182413246 PMC8707162

[32] de Geus JL Wambier LM Bortoluzzi MC. Does smoking habit increase the micronuclei frequency in the oral mucosa of adults compared to non-smokers? A systematic review and meta-analysis. Clin Oral Investig. 2018;22:81–91. 10.1007/s00784-017-2246-4 29063385

[33] Borba TT Molz P Schlickmann DS. Periodontitis: genomic instability implications and associated risk factors. Mutat Res Genet Toxicol Environ Mutagen. 2019;840:20–3. 30857729 10.1016/j.mrgentox.2019.01.005

[34] Nersesyan A Kundi M Atefie K. Effect of staining procedures on the results of micronucleus assays with exfoliated oral mucosa cells. Cancer Epidemiol Biomarkers Prev. 2006;15:1835–40. 17035390 10.1158/1055-9965.EPI-06-0248

[35] Sawhney T Manaktala N Pralhad S. Assessment and comparison of nuclear changes seen in gingivitis and periodontitis using fluorescent microscopy. Rev Esp Patol. 2019;52:208–13. 31530403 10.1016/j.patol.2019.03.002

[36] Argentin G Cicchetti R. Genotoxic and antiapoptotic effect of nicotine on human gingival fibroblasts. Toxicol Sci. 2004;79:75–81. 14718647 10.1093/toxsci/kfh061

[37] Silva RC Neris MA Lopes MA. Chromosomal damage and nuclear alterations in exfoliated cells of the gingival epithelium of individuals with chronic moderate periodontitis. J Biol Med Sci. 2017;16:19–24.

[38] Karatas O Balci Yuce H Tulu F. Evaluation of apoptosis and hypoxia-related factors in gingival tissues of smoker and non-smoker periodontitis patients. J Periodontal Res. 2020;55:392–9. 31854460 10.1111/jre.12723

[39] Goncalves RB Coletta RD Silverio KG. Impact of smoking on inflammation: overview of molecular mechanisms. Inflamm Res. 2011;60:409–24. 21298317 10.1007/s00011-011-0308-7

[40] Souto GR Segundo TK Costa FO. Effect of smoking on Langerhans and dendritic cells in patients with chronic gingivitis. J Periodontol. 2011;82:619–25. 21054228 10.1902/jop.2010.100488

[41] Orbak R Erciyas K Kaya H. Flow-cytometric analysis of T-lymphocyte subsets after different treatment methods in smokers and non-smokers with chronic periodontitis. Int Dent J. 2003;53:159–64. 12873113 10.1111/j.1875-595x.2003.tb00741.x

[42] Rezavandi K Palmer RM Odell EW. Expression of ICAM-1 and E-selectin in gingival tissues of smokers and non-smokers with periodontitis. J Oral Pathol Med. 2002;31:59–64. 11896824 10.1046/j.0904-2512.2001.joptest.doc.x

[43] Seyedmajidi M Keshavarzi P Bijani A. A histopathological study of smoking on free gingiva in patients with moderate to severe periodontitis. Caspian J Dent Res. 2013;2:39–45.

[44] Lindeboom JA Mathura KR Harkisoen S. Effect of smoking on the gingival capillary density: assessment of gingival capillary density with orthogonal polarization spectral imaging. J Clin Periodontol. 2005;32:1208–12. 16268996 10.1111/j.1600-051X.2005.00854.x

[45] Kumar V Faizuddin M. Effect of smoking on gingival microvasculature: a histological study. J Indian Soc Periodontol. 2011;15:344–8. 22368357 10.4103/0972-124X.92566 PMC3283930

[46] Sonmez S Canda T Ozkara E. Quantitative evaluation of the vasculature and fibronectin localization in gingival connective tissue of smokers and non-smokers. J Periodontol. 2003;74:822–30. 12886992 10.1902/jop.2003.74.6.822

[47] Konopacka M. Effect of smoking and aging on micronucleus frequencies in human exfoliated buccal cells. Neoplasma. 2003;50:380–2. 14628093

